# Extracts of *Artemisia ciniformis* Protect Cytotoxicity Induced by Hydrogen Peroxide in H9c2 Cardiac Muscle Cells through the Inhibition of Reactive Oxygen Species

**DOI:** 10.1155/2013/141683

**Published:** 2013-12-04

**Authors:** Mahdi Mojarrab, Maryam Jamshidi, Farahnaz Ahmadi, Ellahe Alizadeh, Leila Hosseinzadeh

**Affiliations:** ^1^Novel Drug Delivery Research Center, School of Pharmacy, Kermanshah University of Medical Sciences, Kermanshah 6734667149, Iran; ^2^Student Research Committee, Kermanshah University of Medical Sciences, Kermanshah 6734667149, Iran

## Abstract

*Objective*. *Artemisia ciniformis* (Asteraceae) and *A. biennis* are two of 34 *Artemisia* species growing naturally in Iran. In this study we investigated whether different extracts of *A. ciniformis* and *A. biennis* have protective effect against hydrogen peroxide-induced cytotoxicity in rat cardiomyoblast cells (H9c2). *Method*. The dried and ground aerial parts of these two species were extracted successively using petroleum ether (40–60), dichloromethane, ethyl acetate (EA), ethanol (EtOH) and ethanol : water (1 : 1) by maceration method. To evaluate whether different extracts of *A. ciniformis* and *A. biennis* protect cardiomyoblast H9c2 cells from H_2_O_2_ cytotoxicity, we examined the direct cytotoxic effect of H_2_O_2_ on H9c2 cells in the presence and absence of different extracts. After then, cell viability was measured by MTT assay. *Results*. H_2_O_2_ induced cytotoxicity in a concentration dependent manner. The IC_50_ value was 62.5 **μ**M for 24 h exposure. However, pretreatment of cells with various concentrations of EA, EtOH, and EtOH/wt extract of *A. ciniformis* protected cells from H_2_O_2_-induced cytotoxicity. Moreover, pretreatment with EA, EtOH and EtOH/wt extracts of *A. ciniformis* lead to a decrease in the reactive oxygen species (ROS) generation. Taken together our observation indicated that nontoxic concentration of different extracts of *A. ciniformis* has protective effect on H_2_O_2_-induced cytotoxicity in H9c2 cells.

## 1. Introduction

*Artemisia biennis *Willd. and *A. ciniformis *Krasch. & Popov ex Poljakov. (Compositae) grow wildly in Iran [1]. Analysis of the essential oils from the aerial parts of *A. biennis *growing in Iran and western Canada revealed the presence of camphor and [E] beta-farnesene as the major constituents, respectively [2, 3]. Myrcene [4] and davanone [5] have been reported as the main constituent in the aerial parts oils of *A. ciniformis*

Cytotoxicity of some fractions of *A. biennis* and *A. ciniformis *as well as significant effects of ethanolic extracts of the species on *in vitro *leishmanicidal activity have been proved [6–8]. Iranshahi et al. [9] reported the presence of high amounts of sesquiterpene lactonesin *A. ciniformis*. Another study showed that antioxidant activity and total phenolic content of hydroethanolic extract of *A. biennis *were higher than those of other extracts [10].

Oxidative stress corresponds to an imbalance between the rate of oxidant production and degradation. It causes numerous biological effects ranging from alternation in signal transduction and gene expression to mutagenesis and finally cell death. It is well known that oxidative stress plays a significant role in the pathogenesis of heart dysfunctions [11]. In our previous study we evaluated the antioxidant activity and total phenolic content of different extracts of *A. biennis* using cell free systems [10]. In the current, study we aimed to examine the effects of *A. biennis *and *A. ciniformis *extracts on hydrogen peroxide (H_2_O_2_)-induced cytotoxicity and oxidative stress in H9c2 cardiomyoblast cells.

## 2. Material and Methods

### 2.1. Reagents and Chemicals

Hydrogen peroxide H_2_O_2_, 3-(4,5-dimethylthiazol-2yl)-2,5-diphenyltetrazolium bromide (MTT), 2,5 dichlorofluorescin diacetate (DCF-DA) were bought from Sigma Aldrich (St Louis, MO, USA). Cell culture medium, penicillin-streptomycin, and fetal bovine serum (FBS) were purchased from Gibco (Gibco, Grand Island, NY, USA). All the solvents used for extraction were purchased from Caledon (Ontaria, Canada) and Scharlau (Sentmenate, Spain).

### 2.2. Plant Material

Aerial parts of *A. ciniformis *Krasch. & Popov ex Poljakov. and *A. biennis *Willd. were collected from Tandoureh national park and Zoshk, respectively (Razavi Khorasan province, Iran), in September 2010. Samples were identified by Dr Valiollah Mozaffarian (Research Institute of Forest and Rangelands, Tehran, Iran). The voucher specimen (Nos. 12569 and 12570) have been deposited in the herbarium, Department of Pharmacognosy, Faculty of Pharmacy, Mashhad University of Medical Sciences, Mashhad, Iran.

### 2.3. Preparation of Extracts and Fractions

The dried powdered aerial parts (80 g) of *A*. *biennis *and *A*. *ciniformis *were extracted with petroleum ether (40–60) (PE), dichloromethane (DCM), ethyl acetate (EA), ethanol (EtOH) and ethanol-water (1 : 1 v/v) (EtOH/wt), respectively (Sequential maceration with ca. 3 × 0.8 L of each solvent). The extracts were filtrated with filter paper and dried using rotary evaporator at a reduced pressure at a temperature below 45°C to yield 4.30, 5.60, 0.39, 1.28, and 8.10 g of each extract for *A. biennis* and 4.13, 9.66, 0.29, 2.54, and 16.08 g for *A. ciniformis*, respectively.

### 2.4. Cell Culture Conditions

Cardiac H9c2 cells are a clonal heart muscle cell line originated from embryonic rat hearts that presents many cardiomyocyte phenotypes [12]. The H9c2 cells maintained in Dubblico modified Eagle's medium (DMEM ATCC) with 10% (V/V) heat inactivated FBS, penicillin G (100 U/mL) and streptomycin (100 mg/mL) at 37°C in 95% CO_2_ humified incubator. The medium was changed 2-3 days and subcultured when the cell population density reached to 70–80% confluence. Cells were seeded at an appropriate density according to each experimental design.

### 2.5. Cell Viability Assay

Cellular toxicities of hydrogen peroxide and different extracts of *A. biennis* and *A. ciniformis *were analysed in H9c2 cells using MTT methods. Four sets of experiments were performed at standard culture conditions: (1) untreated control cells, (2) cells were treated with different concentrations of *A*. *biennis* and *A*. *ciniformis *(10–50 *μ*g/mL), (3) cells were treated with different concentrationsof hydrogen peroxide (25–250 *μ*M), and (4) cells were pretreated with different concentrations of extracts for 24 h, then medium was changed and cells were treated with IC_50_ concentration of hydrogen peroxide for another 24 h. Viability of cells were analyzed using MTT methods. Briefly, after treatment, 20 *μ*L of a 5 mg/mL MTT solution was added to each well. After 2 h incubation, the medium was carefully aspirated and the purple formazan crystals were solubilized with 100 *μ*L DMSO. Optical density was measured at 570 nm (reference wavelength 630 nm) in a microplate reader (Bio-Tek, ELX 800, USA). The absorbance of the untreated culture was set at 100%.

### 2.6. Determination of Intracellular ROS

Intracellular ROS levels were examined using DCF-DA. DCF-DA is a nonfluorescent lipophilic ester that easily crosses the plasma membrane. Into the cytosol the acetate group is rapidly removed by unspecific esterases. The oxidation of this molecule to the fluorochrome DCF results in green fluorescence. The intensity of this fluorescence is generally considered to reflect the level to which ROS are present [12].

After seeding for 24 h, H9c2 cells were washed with PBS buffer (pH 7.4). The cells pretreated with test samples for 24 h were then treated with H_2_O_2_ for an additional 24 h. After washing with PBS, the cells were incubated with 20 *μ*L DCF-DA at 37°C for 30 min. The percentage of DMSO insolution did not exceed from 0.5%. After incubation, cells were lysed with Triton X-100. The fluorescence was measured at an excitation wavelength of 488 nm and an emission wavelength of 528 nm using a fluorescence microplate reader (BioTek, H1M, USA).

### 2.7. Statistical Analysis

Each experiment was performed at least three times and the results were presented as mean ± S.E.M. One-way analysis of variance (ANOVA) followed by Turkey's test was used to compare the differences between means. A probability value of *P* < 0.05 was considered to be statistically significant.

## 3. Results

### 3.1. Cell Viability after Exposure to H_2_O_2_, *A. biennis,* and *A. ciniformis *Extracts Alone

**Figure 1:**
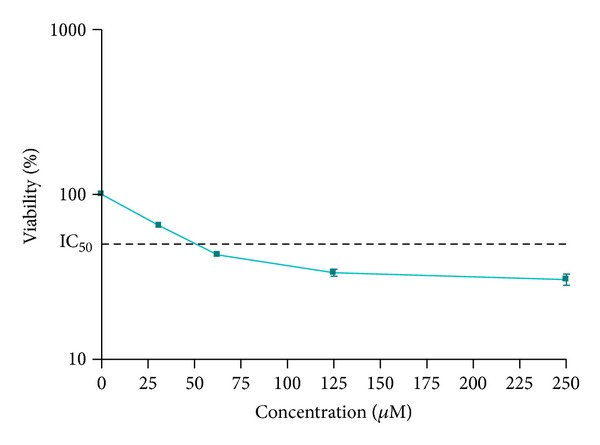
The effect of H_2_O_2_ on H9c2 cell viability. The cell viability was determined by MTT assay as described in material and methods. Data are expressed as the mean ± SEM of three separate experiments (*n* = 6).

The viability of H9c2 cardiomyoblast cells was evaluated after 24 h exposure to different concentrations of H_2_O_2_. Cell viability was evaluated by the MTT method. As shown in Figure 1, H_2_O_2_-induced cytotoxicity was dose dependent. The mean ± SEM IC_50_ value was 62.5 ± 0.034 *μ*M for 24 h exposure to H_2_O_2_. In order to set extracts at concentrations which are nontoxic to cells but could prevent H_2_O_2_-induced cytotoxicity, we also examined the effects of different concentrations of *A. biennis* and *A. ciniformis *extracts on cell viability in H9c2 cells.

**Figure 2:**
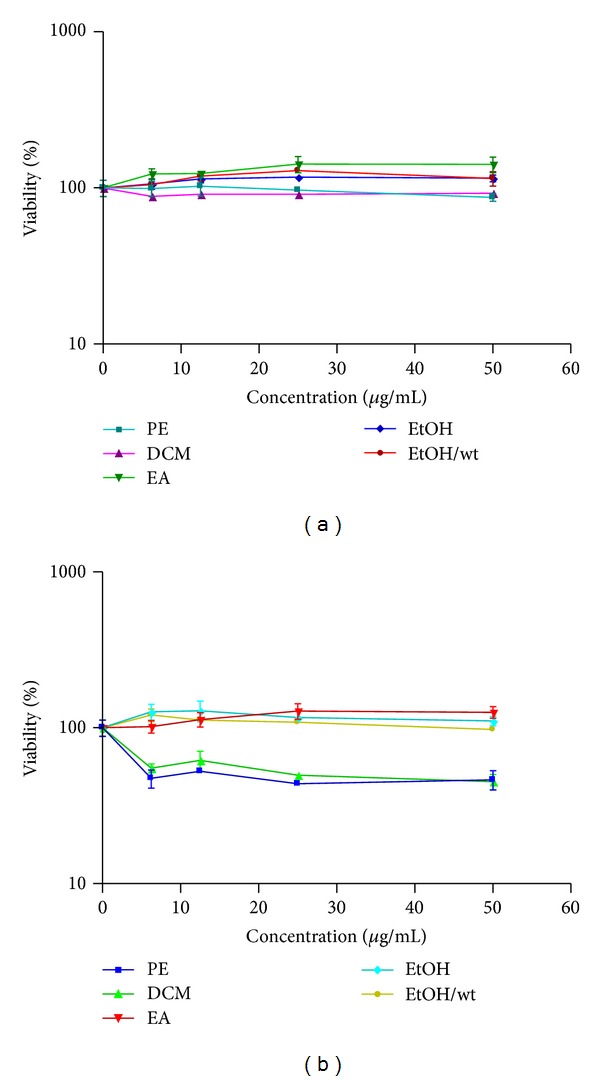
Cell viability of H9c2 cells after exposure to (a) *A. biennis *and (b) *A. ciniformis *Cells were treated with different concentration of extracts for 24 h. The cell viability was determined by MTT assay. Data are expressed as the mean ± SEM of three separate experiments (*n* = 6).

Figure 2 clearly revealed that 24 h treatment with PE, DCM, EA, EtOH, and EtOH/wt extracts of *A. biennis *had no cytotoxic effect at the concentrations up to 50 *μ*g/mL, while 24 h exposure to DCM and PE extracts of *A. ciniformis *induced dose response cytotoxicity.

### 3.2. Effect of Pretreatment with Different Extracts of *A. biennis *and *A. ciniformis *on H_2_O_2_ Induced Cell Death

**Figure 3:**
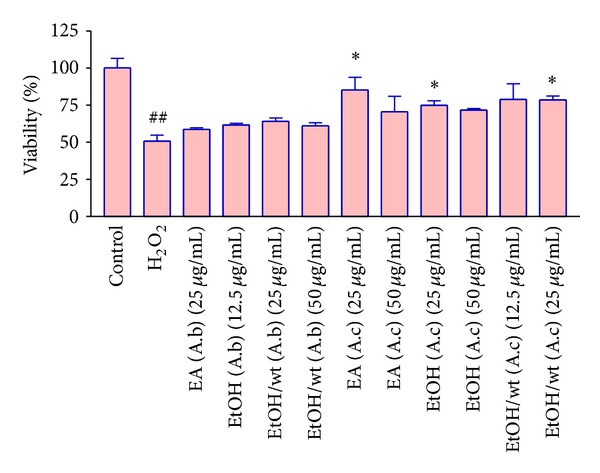
The effect of different extracts of *A*. *biennis *and *A*. *ciniformis *on H_2_O_2_-induced cytotoxicity in H9c2 cells. Cell pretreated with different extracts of *A. biennis *and *A. ciniformis *for 24 h before exposure to 62.5 *μ*M of H_2_O_2_. Data are expressed as the mean ± SEM of three separate experiments (*n* = 6). ^##^ *P* < 0.01 versus control, *P < 0.05, versus H_2_O_2_ treated cells.

For evaluation of effect of pretreatment with different extracts on H_2_O_2_ induced cytotoxicity, H9c2 cells were pretreated for 24 h with nontoxic concentrations of extracts, then the medium was changed and cells treated with IC_50_ concentration (62.5 mM) of H_2_O_2_ for another 24 h. As shown in Figure 1, H_2_O_2_ treatment significantly decreased cell viability to 50 ± 2.2% of control. Adding EA, EtOH, and EtOH/wt extracts of A. *ciniformis* (25 *μ*g/mL) before H_2_O_2_ treatment increased the cell viability to 76 ± 4.53, 72 ± 1.25 and 82 ± 3.21% of control, respectively (Figure 3). Other extracts were not able to protect H9c2 cells against H_2_O_2_-induced cytotoxicity.

### 3.3. Effect of EA, EtOH, and EtOH/wt Extracts of *A. ciniformis *on ROS Induced by H_2_O_2_ in H9c2 Cardiac Muscle Cells

**Figure 4:**
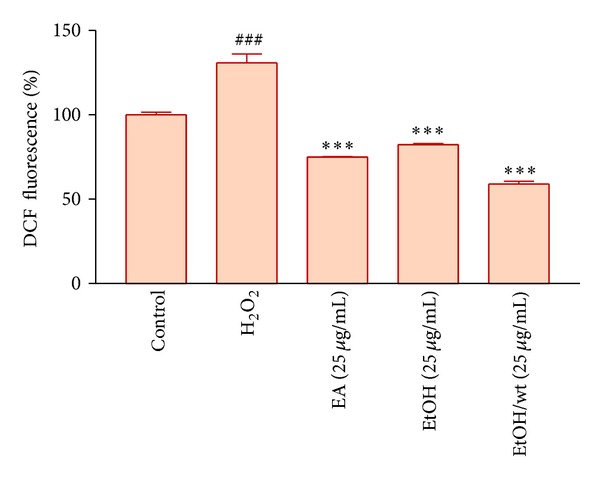
The effect of different extracts of *A. ciniformis *pretreatment on H_2_O_2_-induced ROS generation. Data are expressed as the mean ± SEM of three separate experiments (*n* = 4). ^###^ *P* < 0.001 versus Control, and ***P < 0.001 versus H_2_O_2_-treated cells.

In order to measure oxidative stress induced by H_2_O_2_, fluorescent dye DCF-DA was used to measure ROS generation. As anticipated adding H_2_O_2_ to H9c2 cells caused a significant increase in ROS level. Therefore, cardiomyoblast cells are probably killed due to oxidative stress, since H_2_O_2_ increases intracellular ROS levels. We investigated the inhibitory effect of different extracts on ROS production in the presence of H_2_O_2_. Pretreatment with EA, EtOH, and EtOH/wt extracts of *A. ciniformis *decreased intra cellular ROS levels in H9c2 cells, significantly. These results indicate that the aforementioned extracts have potential for prevention of ROS mediated events (Figure 4).

## 4. Discussion

Oxidative stress is considered to be an important condition to promote cell death in response to a variety of signals and pathophysiological condition [13]. It results from increased formation of ROS and/or decreased antioxidant store. Oxidative stress can be identified in most of the key stages in the pathophysiology of atherosclerosis and the main clinical manifestations of cardiovascular disease [14, 15]. Previous reports demonstrated thatanti-oxidant natural substances including herbal medicines could inhibit ROS generation [16].

In the current study we examined the protective effect of different extracts of *A. biennis *and *A. ciniformis *on the cytotoxicity induced by H_2_O_2_. The obtained results showed that only EA, EtOH, and EtOH/wt extracts of *A*. *ciniformis* are able to protect H9c2 cardiomyoblast cells against H_2_O_2_ cytotoxicity.

Next, it was investigated whether pretreatment with above mentioned extracts had an effect on ROS generation by H_2_O_2_. The obtained results showed that pretreatment with EA, EtOH, and EtOH/wt extracts of *A. ciniformis *leads to a decrease in the ROS generation. One possible explanation for the effect of EA, EtOH, and EtOH/wt extracts of *A*. *ciniformis* on the oxidative stress induced by H_2_O_2_ concerns its polyphenolic content, because it is known that plant-derived polyphenolics are potent antioxidants and free radical scavengers [17].

Despite the fact that hydro ethanolic extract of *A. biennis *showed potent antioxidant effects using free radical scavenging methods it was not able to protect H9c2 cells from cytotoxicity induced by H_2_O_2_ in the current study [10]. This is due to the actual antioxidant activity in physiological conditions such as specific target radicals, localization in different phases and their possible interaction. Therefore, cell free methods may not be sufficient to assessment of antioxidant properties of phytochemicals. Taken together, our data suggested that EA, EtOH, and EtOH/wt extracts of *A*. *ciniformis*, protected cardiomyoblasts against H_2_O_2_-induced cell death by a mechanism believed to be free radical scavenging and/or the inhibition of reactive oxygen species. Thus, EA, EtOH, and EtOH/wt extracts of *A*. *ciniformis* contains principals that may be useful for the prevention and treatment of cardiovascular diseases associated with ROS. Polyphenolics [18], nitrogen containing compounds [19], Polysaccharide fractions [20] and terpenoids [21] are examples of different classes of plant-derived antioxidants. Isolation and characterization of the active and/or major components as well as further studies to determine the molecular mechanisms by which the extracts exert their cardioprotective role are needed.

## References

[1] Mozaffarian V (1998). *A Dictionary of Iranian Plant Names*.

[2] Nematollahi F, Rustaiyan A, Larijani K, Nadimi M, Masoudi S (2006). Essential oil composition of *Artemisiabiennis* willd. and *Pulicariaundulata* (L.) C.A. Mey., two compositae herbs growing wild in Iran. *Journal of Essential Oil Research*.

[3] Lopes-Lutz D, Alviano DS, Alviano CS, Kolodziejczyk PP (2008). Screening of chemical composition, antimicrobial and antioxidant activities of *Artemisia* essential oils. *Phytochemistry*.

[4] Rustaivan A, Masoudi S, Kazemi M (2007). Volatile oils constituents from different parts of *Artemisia ciniformis* Krasch. et M. Pop. ex Poljak and *Artemisia incana* (L.) Druce. from Iran. *Journal of Essential Oil Research*.

[5] Firouzni A, Vahedi H, Sabbaghi F, Bigdeli M (2008). Composition of the essential oil of *Artemisia ciniformis*, *A. kopetdaghensis*, and *A. khorasanica* in Iran. *Chemistry of Natural Compounds*.

[6] Emami A, Zamani Taghizadeh Rabe SH, Ahi A, Mahmoudi M (2010). Study on toxic effects of *Artemisisa* spp. fractions from Iran on human cancer cell lines. *Journal of Zanjan University of Medical Sciences and Health Services*.

[7] Taghizadeh Rabe SZ, Mahmoudi M, Ahi A, Emami SA (2011). Antiproliferative effects of extracts from Iranian *Artemisia* species on cancer cell lines. *Pharmaceutical Biology*.

[8] Emami SA, Rabe SZT, Ahi A, Mahmoudi M (2012). Inhibitory activity of eleven *Artemisia* species from Iran against *Leishmania major* parasites. *Iranian Journal of Basic Medical Sciences*.

[9] Iranshahi M, Emami SA, Mahmoud-Soltani M (2007). Detection of sesquiterpene lactones in ten *Artemisia species* population of Khorasan provinces. *Iranian Journal of Basic Medical Sciences*.

[10] Hatami T, Emami SA, Miraghaee SS, Mojarrab M Total phenolic contents and antioxidant activities of different extracts and fractions from the aerial parts of *Artemisia biennis* Willd.

[11] Herrera B, Murillo MM, Álvarez-Barrientos A, Beltrán J, Fernández M, Fabregat I (2004). Source of early reactive oxygen species in the apoptosis induced by transforming growth factor-*β* in fetal rat hepatocytes. *Free Radical Biology and Medicine*.

[12] Karlsson M, Kurz T, Brunk UT, Nilsson SE, Frennesson CI (2010). What does the commonly used DCF test for oxidative stress really show?. *Biochemical Journal*.

[13] Matés JM, Snchez-Jiménez FM (2000). Role of reactive oxygen species in apoptosis: implications for cancer therapy. *The International Journal of Biochemistry & Cell Biology*.

[14] Sorg O (2004). Oxidative stress: a theoretical model or a biological reality?. *Comptes Rendus*.

[15] Schnabel R, Blankenberg S (2007). Oxidative stress in cardiovascular disease: successful translation from bench to bedside?. *Circulation*.

[16] Brookins Danz ED, Skramsted J, Henry N, Bennett JA, Keller RS (2009). Resveratrol prevents doxorubicin cardiotoxicity through mitochondrial stabilization and the Sirt1 pathway. *Free Radical Biology and Medicine*.

[17] Ishige K, Schubert D, Sagara Y (2001). Flavonoids protect neuronal cells from oxidative stress by three distinct mechanisms. *Free Radical Biology and Medicine*.

[18] Miller AL (1996). Antioxidant flavonoids: structure, function and clinical usage. *Alternative Medicine Review*.

[19] Drolet G, Dumbroff EB, Legge RL, Thompson JE (1986). Radical scavenging properties of polyamines. *Phytochemistry*.

[20] Wang J, Zhang Q, Zhang Z, Li Z (2008). Antioxidant activity of sulfated polysaccharide fractions extracted from *Laminaria japonica*. *International Journal of Biological Macromolecules*.

[21] Das J, Mao AA, Handique PJ (2011). Terpenoid compositions and antioxidant activities of two Indian valerian oils from the Khasi Hills of North-east India. *Natural Product Communications*.

